# Case Report and Review of the Literature: A New and a Recurrent Variant in the *VARS2* Gene Are Associated With Isolated Lethal Hypertrophic Cardiomyopathy, Hyperlactatemia, and Pulmonary Hypertension in Early Infancy

**DOI:** 10.3389/fped.2021.660076

**Published:** 2021-04-16

**Authors:** Katarína Kušíková, René Günther Feichtinger, Bernhard Csillag, Ognian Kostadinov Kalev, Serge Weis, Hans-Christoph Duba, Johannes Adalbert Mayr, Denisa Weis

**Affiliations:** ^1^Department of Pediatric Neurology, Medical School, Comenius University and National Institute of Children's Diseases, Bratislava, Slovakia; ^2^Department of Pediatrics, University Hospital Salzburg, Paracelsus Medical University Salzburg, Salzburg, Austria; ^3^Department of Neonatology, Kepler University Hospital Med Campus IV, Johannes Kepler University Linz, Linz, Austria; ^4^Division of Neuropathology, Department of Pathology and Molecular Pathology, Kepler University Hospital Neuromed Campus, Johannes Kepler University Linz, Linz, Austria; ^5^Department of Medical Genetics, Kepler University Hospital Med Campus IV, Johannes Kepler University Linz, Linz, Austria

**Keywords:** *VARS2* gene, oxidative phosphorylation, mitochondriopathy, hyperlactatemia, lethal hypertrophic cardiomyopathy, pulmonary hypertension

## Abstract

Mitochondriopathies represent a wide spectrum of miscellaneous disorders with multisystem involvement, which are caused by various genetic changes. The establishment of the diagnosis of mitochondriopathy is often challenging. Recently, several mutations of the *VARS2* gene encoding the mitochondrial valyl-tRNA synthetase were associated with early onset encephalomyopathies or encephalocardiomyopathies with major clinical features such as hypotonia, developmental delay, brain MRI changes, epilepsy, hypertrophic cardiomyopathy, and plasma lactate elevation. However, the correlation between genotype and phenotype still remains unclear. In this paper we present a male Caucasian patient with a recurrent c.1168G>A (p.Ala390Thr) and a new missense biallelic variant c.2758T>C (p.Tyr920His) in the *VARS2* gene which were detected by whole exome sequencing (WES). VARS2 protein was reduced in the patient's muscle. A resulting defect of oxidative phosphorylation (OXPHOS) was proven by enzymatic assay, western blotting and immunohistochemistry from a homogenate of skeletal muscle tissue. Clinical signs of our patient included hyperlactatemia, hypertrophic cardiomyopathy (HCM) and pulmonary hypertension, which led to early death at the age of 47 days without any other known accompanying signs. The finding of novel variants in the *VARS2* gene expands the spectrum of known mutations and phenotype presentation. Based on our findings we recommend to consider possible mitochondriopathy and to include the analysis of the *VARS2* gene in the genetic diagnostic algorithm in cases with early manifesting and rapidly progressing HCM with hyperlactatemia.

## Introduction

Mitochondria are organelles responsible for energy production in the form of adenosine triphosphate (ATP) through the oxidative phosphorylation system (OXPHOS). The process of energy production is performed by the mitochondrial respiratory chain and ATP synthase located in the inner membrane of mitochondria (1). Mitochondrial disorders represent a wide spectrum of heterogeneous syndromes predominantly presenting with symptoms as lactic acidosis, hypotonia, developmental delay, failure to thrive, and encephalopathy due to cell energy depletion. Mitochondrial disorders are associated with many causative genes (2–4), which are encoded by mitochondrial DNA (mtDNA) or nuclear DNA (5). Valyl-tRNA synthetase (Val-tRNA) involved in mitochondrial translation (the process of protein synthesis from the information contained in a molecule of messenger RNA – mRNA) is encoded by the *VARS2* gene (6) and catalyzes the amino acid valine's attachment to its tRNA (7). Dysfunction of the protein product is responsible for combined oxidative phosphorylation deficiency 20 (OMIM: # 615917) inherited in an autosomal recessive manner. Rare biallelic variants in the *VARS2* gene have been associated with severe clinical features as mitochondrial encephalomyopathy or encephalocardiomyopathy in 23 affected individuals from 19 families worldwide (1, 4, 6–15). In the present paper we describe a compound heterozygous case with a recurrent c.1168G>A (p.Ala390Thr) and a novel missense variant c.2758T>C (p.Tyr920His) in the *VARS2* gene causing isolated hypertrophic cardiomyopathy, hyperlactatemia, and pulmonary hypertension leading to early death. The pathogenicity of the detected variants is supported by immunohistochemistry, enzymatic analysis, and western blot in a muscle sample of the affected individual. Finally, we compare our findings with the case reports published so far.

## Methods

### Clinical Description

All clinical information about our patient was obtained from medical records retrospectively with the patient's parents' informed consent and was correlated with potential causative mutations detected by whole-exome sequencing (WES).

### Genetic Investigation

Genetic testing was performed from the peripheral blood of the patient. Karyotyping was performed according to a standard cytogenetic protocol. SNP array was performed with Illumina Cyto 850Kv1.1 Bead Chip. In addition, DNA isolated from the patient's peripheral blood lymphocytes was analyzed by whole-exome sequencing. The library was prepared by SureSelect60Mbv6 (Agilent) and paired-end sequenced on a HiSeq 4000 platform (Illumina) with a read-length of 100 bases. In order to align reads to the human genome assembly hg19 Burrows-Wheeler Aligner (BWA, v.0.5.87.5) was applied and detection of genetic variation was performed using SAMtools (v 0.1.18), PINDEL (v 0.2.4t), and ExomeDepth (v 1.0.0). The cut-off for biallelic inheritance was set to <1% allele frequency, for monoallelic inheritance to <0.1%. The size of reference entries was >20,000 exomes in the database at the time of analysis (16). With this approach, 97.3% of the target sequences were covered >20-fold. The detected mutations were verified by Sanger sequencing, followed by examining the DNA from peripheral blood of the patient's parents.

### Muscle Biopsy

The biopsy of the vastus lateralis muscle was performed under general anesthesia according to standard procedures. Muscle tissue was cut into several pieces for formalin fixation, glutaraldehyde fixation, and rapid freezing in isopentane cooled in liquid nitrogen for subsequent analyses.

#### Histopathology

The histopathology and the enzyme histochemical stain analysis encompassed the following stains: Haematoxylin and Eosin (H&E), Elastica van Gieson (EvG), Congored, ATPase at pH 9,4, pH 4,3 and 4,2, reduced nicotinamide adenine dinucleotide-tetrazolium reductase (NADH), Gomori trichrome, cytochrome c oxidase (COX), succinate dehydrogenase (SDH), acid phosphatase, periodic acid-Schiff (PAS), Sudan black, myoadenylate deaminase (MAD), phosphofructokinase (PFK), phosphorylase, and acetylcholinesterase (ACE). The immunohistochemical analyses included the following antibodies: CD3, CD20, CD8, CD4, CD45, CD79a, CD68, HLA-DRII, beta-spectrin, alpha-sarcoglycan, beta-sarcoglycan, gamma-sarcoglycan, delta-sarcoglycan, dystrophin (N-terminus), dystrophin (C-terminus), dystrophin (rod domain), dysferlin, titin, emerin, telethonin, POMT1, myotilin, lamin A/C, caveolin-3, actinin, laminin2, collagen VI, desmin, myosin fast, myosin slow, myosin neonatal, membrane attack complex, vimentin, and utrophin at appropriate dilutions.

#### Enzymatic Analysis

Muscle 600 × g homogenates were used for determination of enzymatic activities of the OXPHOS complexes. Enzyme activities of the OXPHOS complexes were determined as previously described (17). The rotenone-sensitive complex I activity was measured spectrophotometrically as NADH/decylubiquinoneoxidoreductase at 340 nm. The enzyme activities of citrate synthase, complex IV (ferrocytochrome c/oxygenoxidoreductase), and oligomycin-sensitive ATPase activity of the F_1_F_O_ ATP synthase (complex V) were determined as previously described (18). The whole reaction mixture for the ATPase activity measurement was treated for 10 s with an ultra-sonifier (Bio cell disruptor 250, Branson, Vienna, Austria). The reaction mixture for the measurement of the complex III activity contained 50 mM potassium phosphate buffer pH 7.8, 2 mM EDTA, 0.3 mM KCN, 100 μM cytochrome c, 200 μM reduced decyl-ubiquinol. The reaction was started by addition of the 600 g homogenate. After 3–4 min the reaction was inhibited with 1 μM antimycin A. All spectrophotometric measurements (Uvicon 922, Kontron, Milan, Italy) were performed at 37°C.

#### Substrate Oxidation Analysis

Muscle 600 × g homogenate was incubated with different ^14^C-labeled substrates (Table 1) for 20 min at 37°C according to Bookelman et al. (19) in a reaction volume of 50 μl. The reactions were stopped by the addition of 20 μl of 15% HClO_4_ and the ^14^CO_2_ was collected in 1M NaOH-wetted filter paper snippets (Whatman) placed in the screw cap of 2 ml reaction tubes (Sarstedt). After incubation for 15 min on ice, the filter papers were transferred to new 2 ml reaction tubes containing 1 ml of scintillation buffer Ultima Gold (Perkin Elmer) and counted in a scintillation counter (Packard 1600 TR). The scintillation counts of the different substrate combinations were related to the total activities per reaction and calculated as nmol/h/mg protein activity oxidation.

**Table 1:** OXPHOS enzymes activity measurements and substrate oxidation in muscle of the reported patient with *VARS2* deficiency.

**Enzyme**	**mUnit/mg/** **protein**	***N***	**mUnit/mUnit** **CS**	***N***	**mUnit/mUnit** **Complex II**	***N***
Citrate synthase (CS)	198	150–338			3,02**↓**	3,06–5,47
Complex I	**12↓**	28–76	**0,06↓**	0,14–0,35	**0,18↓**	0,46–1,07
Complex I + III	80	49–218	0,41	0,24–0,81	1,22	0,93–2,84
Complex II (CII)	66	33–102	0,33	0,18–0,41		
Complex II + III	171	65–180	**0,86↑**	0,3–0,67	2,61**↑**	1,36–2,49
Complex III	611	304–896	3,09	1,45–3,76	9,32	5,94–12,91
Cytochrome c oxidase	193	181–593	0,97	0,91–2,24	**2,94↓**	4,19–12,05
Complex V	248	86–257	1,25	0,42–1,25	3,78	1,68–3,96
Pyruvate dehydrogenase	20,6	5,3–19,8	**0,104↑**	0,026–0,079	0,314	0,107–0,315
**Substrate oxidation**	**nmol/h/mUnit CS**	***N***	**mUnit/mUnit CS**	***N***	**nmol/h/mUnit Complex II**	***N***
[1-14C]Pyruvate+Malate	600	263–900	3,03	1,54–3,55	9,15	3,57–11,97
[1-14C]Pyruvate+Carnitine	**948↑**	302–856	**4,79↑**	1,65–3,66	14,45**↑**	4,48–14,24
[1-14C]Pyruvate+Malate-ADP	**119↑**	32–102	**0,60↑**	0,21–0,41	1,82	0,69–1,88
[1-14C]Pyruvate+Malate+ CCCP	803	304–889	**4,05**↑	1,31–3,11	12,24	3,55–12,35
[1-14C]Pyruvate+Malate+Atracyloside	**134↑**	19–90	**0,68**↑	0,16–0,55	3,24	1,17–3,61
[U-14C]Malate+Pyruvate+Malonate	509	282–874	2,57	1,56–3,87	7,75	3,69–12,53
[U-14C]Malate+Acetylcarn.+Malonate	517	273–678	2,61	1,16–2,82	7,89	3,8–11,44
[U-14C]Malate+ Acetylcarn.+Arsenite	301	156–378	1,52	0,57–1,52	4,58	2,17–6,14
[U-14C]Glutamate+Acetylcarnitine	162	86–209	0,82	0,35–1,06	2,48	1,4–5,11

*N, normal range; CS, citrate synthase, protein concentration of the patient muscle 600 × g homogenate: **1.64** mg/ml. Values in bold show abnormal values. Arrow down: reduced values compared to healthy controls. Arrow up: increased values compared to healthy controls*.

#### Western Blot

Muscle tissue homogenates from the patient and controls (centrifuged at 600 × g) were analyzed on 10% acrylamide/bisacrylamide gels and transferred to nitrocellulose membranes. The membranes were washed in Tris-buffered saline (TBS) for 5 min, air-dried for 30 min, washed 10 min in TBS, and blocked 1 h at room temperature in 1x western blocking solution in TBS-T (Roche, Mannheim, Germany). After washing with TBS-Tween 20 (0.5%; TBS-T), the membranes were incubated with the primary antibody diluted in 1x western blocking solution in TBS-T. The indicated primary antibody dilutions and incubation times were used for western blot analysis: polyclonal rabbit VARS2 (1:1,000, o/n, 4°C; Proteintech), monoclonal mouse NDUFS4 (1:1,000, 1 h, room temperature; Abcam), SDHA (1:2,000, 1 h, room temperature; Abcam), UQCRC2 (1:1,500, 1 h, room temperature; Abcam); MT-CO2 (1:1,000, 1 h, room temperature; Abcam), ATP5F1A (1:2,000, 1 h, room temperature; Abcam), VDAC1 (1:2,000, 1 h, room temperature; Abcam), CS (1:3,000, 1 h, room temperature; THP), GPI (1:800, 1 h, room temperature; Santa Cruz). Membranes were incubated with secondary antibodies labeled polymer horseradish peroxidase-(HRP) 1:100 (EnVisionkit, Dako) at room temperature. Detection was carried out with Lumi-Light PLUSPOD substrate (Roche).

#### Immunohistochemistry

Muscle tissue was stained as previously described (20–23). For IHC, the following antibodies were used: complex I subunit NDUFB8 (rabbit polyclonal, 1:500; Abcam, Cambridge, UK), complex II subunit SDHA (mouse monoclonal, 1:2,000; Abcam, Cambridge, UK), complex III subunit UQCRC2 (mouse monoclonal, 1:1,500; Abcam, Cambridge, UK), complex IV subunit MT-CO1 (mouse monoclonal, 1:1,000; Abcam, Cambridge, UK), complex V subunit ATP5F1A (mouse monoclonal, 1:2,000; Abcam, Cambridge, UK), and VDAC1 (mouse monoclonal, 1:3,000; Abcam, Cambridge, UK). All antibodies were diluted in Dako antibody diluent with background-reducing components (Dako, Glostrup, Denmark). For antigen retrieval, the sections were immersed for 45 min in 1 mM EDTA, 0.05% Tween-20, pH 8, at 95°C. Muscle tissue sections were incubated for 60 min with the above-mentioned primary antibodies. Staining was done with the DAKO envision kit.

## Results

### Case Report – Clinical Features of the Patient

The male patient was born at term (at 39+3 weeks of gestation) by vaginal delivery using vacuum extraction, as the first child of non-consanguineous Caucasian parents. Due to the prenatally suspected aortic stenosis, the patient was immediately transferred to the Neonatal Intensive Care Unit. Birth weight (3140 g; 21st percentile), birth length (51 cm; 48th percentile), head circumference (33.5 cm; 17th percentile), Apgar scores 9/10/10, and pH from the umbilical artery were normal (pH: 7.27). The newborn had marks after vacuum extraction over the parietal region (cephalohematoma); muscle tone was normal, and no dysmorphic features were present. Postnatal echocardiography showed borderline width of the isthmus and aortic arch (but still within normal range), bicuspid aortic valve, and an open ductus arteriosus Botalli. No surgery or prostaglandin treatment was necessary, but the patient developed a need for oxygen therapy. On the third day after birth blood gas analysis showed an uncompensated metabolic acidosis with hyperlactatemia: plasma lactate: 6.7 mmol/l (normal range <2.1 mmol/l), pH: 7.28 (N: 7.35–7.45), pCO_2_: 42.1 mmHg (N: 35–45 mmHg), ${\text{HCO}}_{3}^{-}$: 18.2 mmol/l (N: 21–28), Base excess: −7.3 (N: 0 ± 3 mmol/l) with normoglycemia (Blood glucose: 59 mg/dl, N: 50–114). Anion gap was slightly elevated (AG: 16.9, N: 7–16). Since an inherited disorder of metabolism was suspected, metabolic screening was performed, which showed repeatedly lactate elevation: preprandial lactate: 6 mmol/l (N: 0.5–1.6), 3-OH-butyrate: 0.1 mmol/l (N: 0.03–0.3) glucose: 57 mg/dl (N: 50–114); postprandial lactate: 4 mmol/l, 3-OH-butyrate: 0.3 mmol/l, glucose: 84 mg/dl; plasmatic alanine elevation: 539.8 μmol/l (N: 116–376) and normal level of lactate in urine (19 mmol/mol creatinine, N 51 (1–156) mmol/mol creatinine). Other parameters (acylcarnitine profile, creatine kinase, aminotransferases, ammonemia, electrolytes, blood count) were normal. Glucose infusion (5 mg/kg/min.) was administered. The patient could have oral intake (suction) and neurological status was normal, with no signs of lethargy or hypotonia. Sonography of the abdomen did not show any pathology, especially no hepatosplenomegaly. Ophthalmologic examination showed normal findings without signs of cataract. The patient developed progressive symptoms of heart failure over the next few days. Echocardiography on the 16th day of life revealed primary pulmonary hypertension and hypertrophic cardiomyopathy of the left heart. Investigation of alpha-glucosidase excluded Pompe disease (19 nmol/mg–normal value). Cardiac catheterization showed pulmonary hypertension (PHT) and hemodynamic failure of the right heart. The myocardial biopsy was contraindicated due to the patient's severe clinical condition. Dobutamine, nitric oxide therapy, sildenafil, and oxygen therapy, with 60% FiO2 was administered. Based on suspicion of a mitochondrial disorder, muscle biopsy from the vastus lateralis muscle was performed for histopathological purposes. No changes for a primary muscle disorder were found. Based on specific enzyme histochemical staining (e.g., combined COX-SDH), no evidence for the presence of a mitochondrial myopathy could be demonstrated. Genetic testing of the *ACAD9* gene associated with mitochondrial complex I deficiency, nuclear type 20 (OMIM: # 611126) did not reveal any pathogenic mutation, and therefore whole-exome sequencing was indicated. Because of lasting suspicion of a mitochondrial disorder, treatment with riboflavin (20 mg/kg/d), coenzyme Q10 (30 mg/kg/d), and continuous intravenous glucose was administered. Under this treatment, lactate levels declined but still were above normal values. Levels were elevated during PHT crises (values during PHT crises were 11–13.3 mmol/l). On day 22, cardiac insufficiency worsened, and a first cardiopulmonary resuscitation was necessary. MRI of the brain and thorax performed on day 23 showed a focal hemorrhage in the right frontal subcortical region. The degree of myelination was appropriate for age. Cardiomegaly was evident. Low pleural effusions on both sides of the thorax and dystelectasia/atelectasia in both lungs were seen. On day 31, because of recurrent PHT crisis, dilatation and stent treatment of the foramen ovale was performed but was not successful. The patient was intubated and sedated with ketamine, midazolam, sufentanil, and myorelaxed. Cardiomyopathy progressed despite intensive care and the patient died on day 47 due to cardiopulmonary failure. A few days after the patient's death, the result of the WES analysis confirmed the state of compound heterozygosity for variants in the *VARS2* gene, supporting the assumption of a mitochondrial disorder in the child. Following the wish of the parents, no autopsy was performed.

### Molecular Genetics Findings

**Figure 1:**
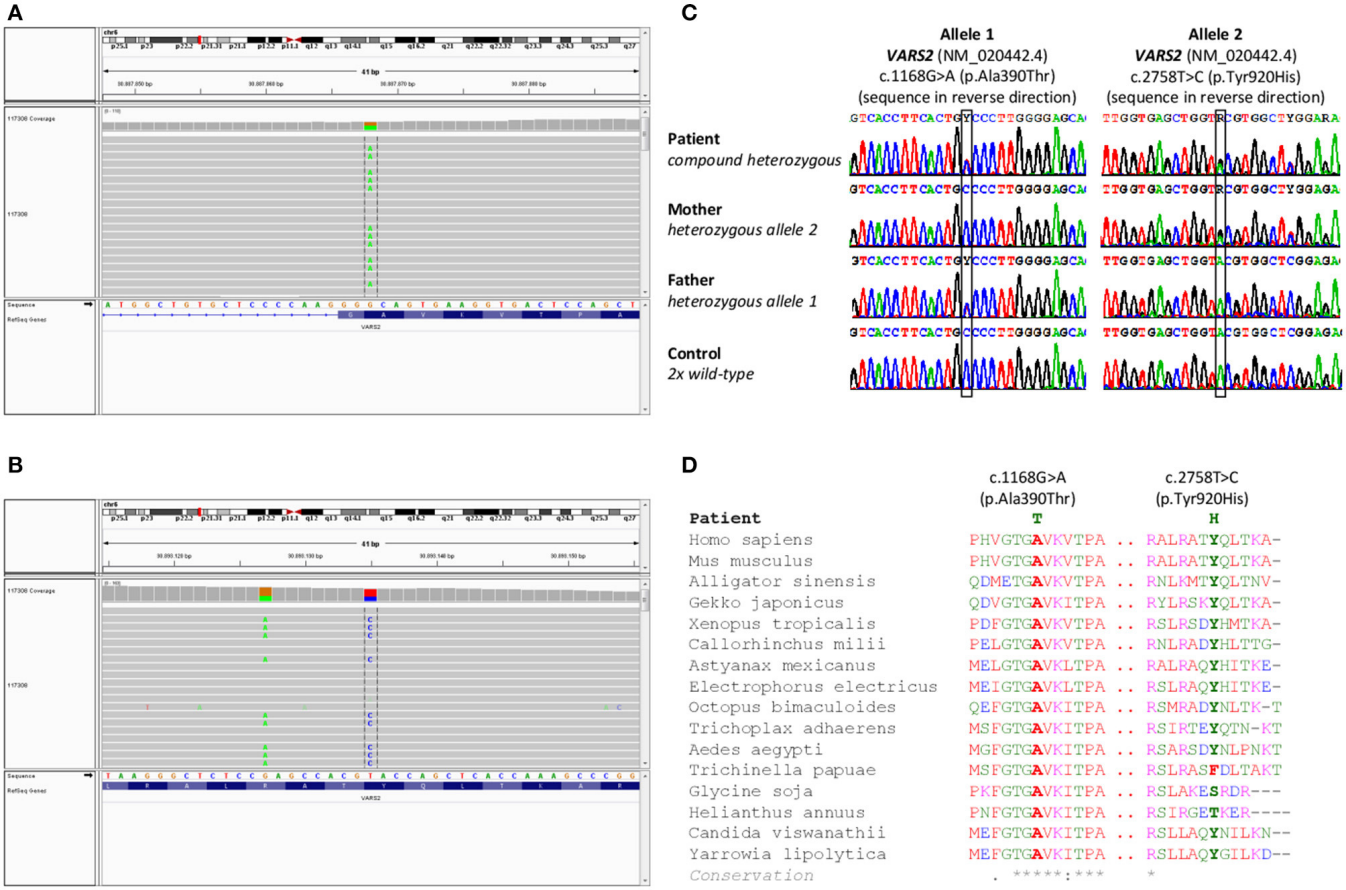
Results of genetic testing. **(A,B)** Whole exome sequencing results in a patient with a finding of biallelic variants in the *VARS2* gene, heterozygous variant c.2758T>C (p.Tyr920His) and heterozygous variant c.1168G>A (p.Ala390Thr)**. (C)** Sanger sequencing confirmed both variants in the patient. Heterozygous variant c.2758T>C (p.Tyr920His) was found in patient's mother and heterozygous variant c.1168G>A (p.Ala390Thr) was found in patient's father. These findings confirmed biallelic position of variants in the patient, **(D)** shows phylogenetic conservation of VARS2 protein (NP 065175.4) in various organisms. Multiple sequence alignment was performed with Clustal Omega (https://www.ebi.ac.uk/Tools/msa/clustalo/).

Cytogenetic examination showed a normal male karyotype 46, XY. Genomic imbalances were ruled out by the SNP array. The whole-exome sequencing revealed the following biallelic variants in *VARS2* gene: allele 1 [NM_020442.6:exon13: c.1168G>A (p.Ala390Thr)] (https://www.ncbi.nlm.nih.gov/clinvar/variation/522814/); allele 2 [NM_020442.6:exon27: c.2758T>C (p.Tyr920His)] (https://www.ncbi.nlm.nih.gov/clinvar/variation/997679/) (Figures 1A,B). The finding was verified by Sanger sequencing. Examination of the parents showed the paternal *VARS2* variant c.1168G>A (p.Ala390Thr) in a heterozygous form and the maternal *VARS2* variant c.2758T>C (p.Tyr920His) in a heterozygous form (Figure 1C). Both variants involve residues that are highly conserved among phylogenetically distant organisms. At position 920, tyrosine is most frequently found in some organisms, also the amino acid phenylalanine, both with a phenyl group. In plants, this position is not conserved with serine or threonine in the alignment. Position 390 is highly conserved (Figure 1D). Position 390 corresponds to the tRNA-synthetase domain and position 920 corresponds to the anticodon binding domain of the protein VARS2 (1, 12). These findings support the pathogenicity of the variants found in our patient.

### Histopathology, Immunohistochemistry, Enzymatic Analysis, Substrate Oxidation, and Western Blot

**Figure 2:**
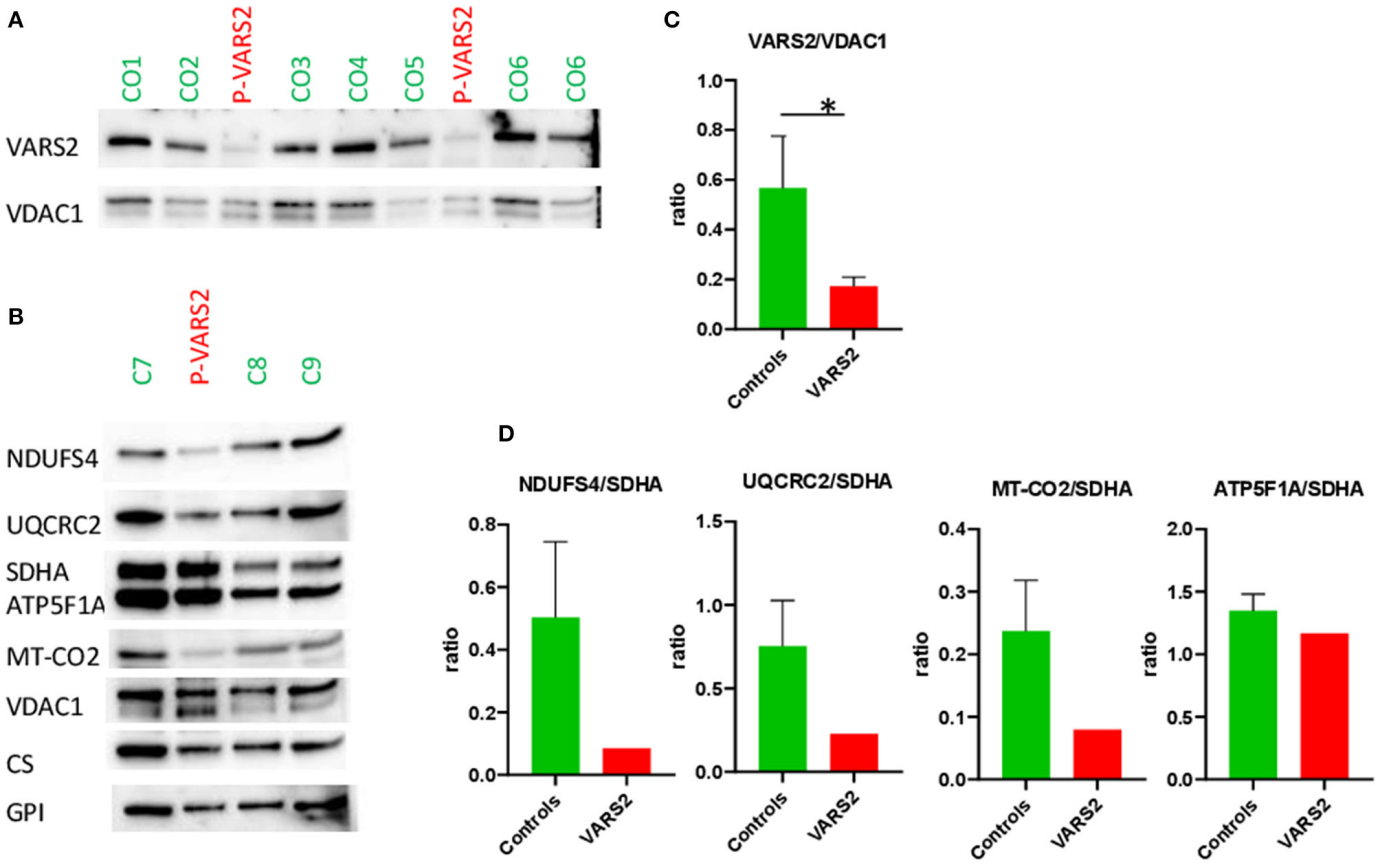
**(A,B)** Western blot analysis of the muscle sample of the patient compared to healthy controls, using antibodies against VARS2, NADH dehydrogenase [ubiquinone] iron-sulfur protein 4 (NDUFS4), ubiquinol-cytochrome c reductase core protein 2 (UQCRC2), succinate dehydrogenase complex flavoprotein subunit A (SDHA), ATP synthase F1 subunit alpha (ATP5F1A), mitochondrially encoded cytochrome c oxidase II (MT-CO2), voltage-dependent anion-selective channel 1 (VDAC1, in outer mitochondrial membrane), citrate synthase (CS), glucosephosphate isomerase (GPI). **(C,D)** Bar graphs show the densitometry of the western blot bands. The intensity of the bands was determined using evaluation software (Imagelab, Biorad). The subunits of the OXPHOS complexes were evaluated in relation to VDAC1, CS, and GPI as loading controls. The results showed reduction of complex I in comparison to SDHA; moderate reduction of complex III and IV in comparison to SDHA and compensatory up-regulation/increase of complexes II and V. The ratios to VDAC1, CS, and GPI are shown in Supplementary Table 1. *p < 0.05.

Western blot analysis revealed a reduction of VARS2 protein amount in muscle homogenates of the affected patient compared to seven controls (Figures 2A,C; Supplementary Table 1). VARS2 was normalized to four loading proteins namely VDAC1, CS, GPI, and SDHA. Residual VARS2 protein levels were between 16 and 34% of the control levels. No obvious histopathological findings pointing toward a mitochondrial disorder were found. The enzymatic analysis showed values in or above the normal range for the mitochondrial marker enzyme citrate synthase (summary of the results is shown in Table 1). Absolute complex I activity was significantly reduced in patient muscle compared to controls. Consistently, the complex I activities normalized to the citrate synthase and complex II were significantly lower in patients muscle (Table 1). Normalized to complex II which is not dependent on mitochondrial translation a downregulation of complex IV was found. An increase in combined normalized complex II-III activity was present. A compensatory upregulation of unaffected complexes is a well-known phenomenon in mitochondrial disorders. In substrate oxidation analysis some activities were in relation to the protein content in the normal range, others were elevated. Also in relation to citrate synthase similar activities were high. In relation to complex II the oxidation rate of pyruvate + carnitine was slightly elevated. These elevated activities are likely due to compensatory upregulation of mitochondria. In contrast, the ratio of the long oxidation path via pyruvate + malate compared to pyruvate + carnitine was somewhat reduced with a value of 0.63 (normal 0.68–1.09). This is consistent with a defect in the respiratory chain, while the reaction from pyruvate to acetyl-carnitine works better. This shortcut of the full oxidation route forms only one NADH, while the whole path creates 4 NADH and 1 FADH_2_. Consistently, western blot analysis revealed a reduction of complexes I, III and IV in patient homogenate compared to controls (Figure 2). The reduction was present compared to several normalization proteins VDAC1 (outer mitochondrial membrane), CS (mitochondrial matrix), GPI (cytosol) and SDHA (inner mitochondrial membrane and independent from mitochondrial translation) (Figure 2; Supplementary Figure 1; Supplementary Table 1). Also in immunohistochemical staining of VDAC1 (Voltage-dependent anion-selective channel 1) and subunits of the OXPHOS complexes in skeletal muscle showed a slight reduction of NDUFB8 (complex I subunit) and UQCRC2 (complex III subunit) in the affected individual compared to controls (Supplementary Figure 2). A compensatory upregulation of complex II was also present in immunohistochemical staining.

## Discussion

Mitochondriopathies associated with mutations in mitochondrial aminoacyl-tRNA synthetases present a wide spectrum of miscellaneous disorders based on various genetic changes. Recently, several mutations in *VARS2* gene were associated with clinical features such as hypotonia, psychomotor delay, encephalopathy, cardiomyopathy, hyperlactatemia, but the correlation between genotype and phenotype remains unclear (1, 15). To date, 19 families with more than 23 affected individuals have been described in the literature worldwide (1, 4, 6–15) whereby c.1100C>T (p.Thr367Ile) is the most common variant in the *VARS2* gene present in 60.8% (14/23) of the published cases (see Table 2). Common features of homozygous carriers for variant c.1100C>T (p.Thr367Ile) include microcephaly, global psychomotor delay, and hypotonia, less often nystagmus, limb spasticity, and difficulties with feeding. In the first 12 months of life, ataxia, dystonic movements, and seizures can occur, which later (from 2 to 4 years) almost always lead to refractory epilepsy and status epilepticus based on mitochondrial encephalopathy (1, 6, 13). Nevertheless, hypertrophic cardiomyopathy (HCM) has never been observed in these patients (eight patients of which five had no HCM, in three cases the results of cardiologic examination are not available) (1, 6, 13, 15). However, in compound heterozygotes (for variant c.1100C>T (p.Thr367Ile) and another, or two other pathogenic variants, hypertrophic cardiomyopathy was present in 14/15 cases (1, 4, 7–12, 14). These additional findings support Bruni et al. (1) assumption that “*c.1100C*>*T variant could have a lesser effect to the heart.”* In the present paper we describe a compound heterozygous case with a recurrent c.1168G>A (p.Ala390Thr) and a novel missense variant c.2758T>C (p.Tyr920His) in the *VARS2* gene. Position 390 corresponds to the tRNA-synthetase domain and position 920 corresponds to the anticodon binding domain of the protein VARS2 (1, 12). Both variants show a relatively high level of phylogenetic conservation (Figure 1D). The p.Ala390Thr was reported in ClinVar four times (Accession number: VCV000522814.3). Bruni et al. (1) published a case with a homozygous p.Ala390Thr underlining its pathologic relevance. However, we for the first time proved pathogenicity of this obviously recurrent variant with biochemical and immunohistochemical methods. Here we show that the recurrent p.Ala390Thr in combination with a novel missense variant causes a severe reduction of VARS2 protein amount. In contrast with published data, in our case, HCM was a leading feature accompanied by hyperlactatemia and pulmonary hypertension, which led to early death (on 47th day of life) without any other accompanying signs. We see a certain similarity in the case of a patient with pathogenic *VARS2* mutations c.643C>T (p.His215Tyr) and c.1354A>G (p.Met452Val) who presented with poor sucking at birth, poor motor activity, hyporeflexia, hypertonia, persistent pulmonary hypertension of the newborn (PPHN), metabolic acidosis, severe lactic acidosis, and hypertrophic cardiomyopathy (12). The patient died on the 16th day of life due to cardiac arrest due to pulmonary hypertension and hypertrophic cardiomyopathy. Our patient had a normal neurological assessment with no hypotonia and no signs of encephalopathy, neither clinically nor on brain MRI on the 23rd day of life; this finding contrasts with published data (1, 15). Finally, we correlated published histopathological findings of skeletal muscle tissue with negative findings in our patient. Five of 13 patients reported by Bruni et al. (1) underwent muscle biopsy (quadriceps muscle). Two patients showed normal histopathologic findings, one case had mild unspecific myopathic changes with COX-deficient/SDH-positive fibers, and one patient showed signs of neurogenic atrophy. The fifth patient showed at the electron microscopy level mitochondria that were relatively decreased in number and size, with mostly unremarkable morphology, although some were distorted or atypical (with atypical cristae). The data from San Millan et al. (8) demonstrate an uneven involvement of muscles with predominant involvement of myocardium and diaphragm. Our patient had no obvious histopathological findings pointing toward a mitochondrial disorder in vastus lateralis muscle. Unfortunately, a myocardial biopsy was contraindicated in our patient due to the patient's serious clinical condition and an autopsy was not done following the wish of the patient's parents. It would be interesting to evaluate the lactate peak by MR spectroscopy and determine lactate and alanine in cerebrospinal fluid (1, 6, 11, 14), but these were unfortunately not performed during the patient's life. OXPHOS activity was evaluated in eight patients from Bruni et al. (1), Diodato et al. (6) San Millan et al. (8), and Begliuomini et al. (15). However, OXPHOS activity was decreased only in six of them (the first patient had low complex IV activity, the second patient had combined complex I + complex IV deficiencies, the third patient had low complex IV activity, the fourth had low complex I activity, the fifth had low complex IV activity, and the sixth showed reduced complex I and III activity). In our case the results showed a strong reduction of complex I, a moderate reduction of complex III and IV, and compensatory upregulation in complex II and V. A review of all published patients so far is shown in Table 2. Clinical manifestations, disease severity, and life expectancy vary significantly between published cases. We assume that the different phenotypic manifestations in the reported cases could result from the variable expression of valyl-tRNA synthetase in different tissues at different time points after birth, and/or due to unknown epigenetic effects, but further investigations in this field are needed.

**Table 2:** Review of published cases and new patient with *VARS2* deficiency – clinical characteristics, laboratory and molecular genetics findings [modified after (1)].

**Patient/** **family**	**References**	**Sex**	**Mutation**	**Ethnicity**	**Current age/death**	**Onset**	**Hearth**	**Neurological signs**	**MRI + spectroscopy/other features**	**Lab tests/** **Lactate**	**OXPHOS** ** muscle**
**Our caseP24/F20**	**Kuš**í**ková et al., this study**	**M**	**c.1168G>A** **c.2758T>C**	**Caucasian** **(Austrian)**	**Death 47 d**.	**From birth**	**HCM**	**Normal**	**Normal MRI of the brain** **+ PPHN**	**Plasma(4–13 mmol/l)**	**↓↓ CI** **↓ CIII+CIV ↑ CII+CV**
P1/F1	Bruni et al. (1)	F	Homozygous c.1100C>T	Caucasian (Polish)	Alive at 5 y.	From birth	No HCM	Hypotonia, poor coordination, develompmental delay, seizures	Hyperintensity in the periventricular white matter bilaterally, cerebral atrophy; small lactate peak at MRS	N/A	N/A
P2/F2	Bruni et al. (1)	F	c.2557-2A>G c.1100C>T	Caucasian	Death at 3,5 m.	From birth	HCM	Hypotonia, hyporeflexia, exag. startle, staring episodes, vocal cord paralysis	Diffuse cerebral and cerebellar atrophy, focal gliosis around the Sylvian fissures bilaterally, cerebellum, scattered areas of cortical restricted diffusion, subtle thalamic restricted diffusion bilaterally, large lactate peak at MRS	Plasma (1.7–8.9 mmol/l) and urinaryelevation	Normal
P3/F3	Bruni et al. (1)	M	c.1546G>T c.2239G>A	Jewish comunity	Death at 19 m.	From birth	HCM	Hypotonia, severe stridor, poor sucking, hypertonia of the lower limbs	N/A	Plasma(3.5–4 mmol/l)and urinaryelevation	↓ CIV
P4/F4	Bruni et al. 2018 (1)	F	c.1100C>T c.1150G>A	Italian	Death at 5 m.	From birth	HCM	Hypotonia, feeding difficulties and psychomotor delay	Cerebellar atrophy, corpus callosum hypotrophy	Plasma(4.2 mmol/l)and CSF(3 mmol/l;Nv <2.3 mmol/l)	Normal activities
P5/F5	Bruni et al. (1) and Taylor et al. (4)	M	c.1135G>A c.1877C>A	British	Alive at 18 y.	First few months	mild concentric ventricular hypertrophy	Developmental delay, ptosis and ophtalmoparesis, generalized epilepsy, fatigue, proximalweakness, dyspraxia	Symmetrical bilateral basal ganglia calcification, symmetrical increased T2 signal in the peri-trigonal white matter	N/A	↓ CI+CIV
P6/F6	Bruni et al. (1) and Pronicka et al. (9)	M	c.1100C>T c.1490G>A	Polish	Death at 3 m.	Birth	HCM	Hypotonia, stridor andrespiratory failure, limbsspasticity	N/A	Plasma (4.4–8.7 mmol/l)	↓CIV
P7/F6	Bruni et al. (1) and Pronicka et al. (9)	M	c.1100C>T c.1490G>A	Polish	Death at 9 y.	From birth	HCM	Hypotonia, stridor andrespiratory failure, limbsspasticity, epilepsy	Hypoplasia of vermis, mild cerebral atrophy, small symmetric hyperintense changes in thalamus and septum pellucidum	Plasma (2.9–10.6 mmol/l)	N/A
P8/F7	Bruni et al. (1)	M	Homozygous c.1258G>A	Mexican	Death at 9 d.	From birth	HCM	Hypotonia, feeding difficulty	N/A	Plasma elevation	N/A
P9/F7	Bruni et al. (1)	F	Homozygous c.1258G>A	Mexican	Death at 3 m.	From birth	HCM	Hypotonia, feeding difficulty	N/A	Plasma elevation	N/A
P10/F8	Bruni et al. (1)	M	Homozygous c.1258G>A	Mexican	Alive at 3 m.	From birth	HCM	Hypotonia, feeding difficulty, respiratory distress, developmental delay, epilepsy	N/A	Plasma elevation	N/A
P11/F9	Bruni et al. (1)	F	Homozygous c.1100C>T	Afganistan	Death at 7 y.	From 1st month	N/A	Severe hypotonia, feedingdifficulty, psychomotorretardation, nystagmus, intractable epilepsy	Cerebellar atrophy (cerebellar hemispheres + vermis), signal intensity in dentate nuclei, signal intensity and mild atrophy in thalami, corpus callosum slightly thin	Plasmanormal(2.3 mmol/l)	N/A
P12/F9	Bruni et al. (1)	F	Homozygous c.1100C>T	Afganistan	Death at 8 y.	From 1st month	N/A	Hypotonia, feeding difficulty (gastrostomy), psychomotor retardation, limb spasticity, intractableepilepsy	Cerebellar atrophy, signal intensity in dentatenuclei and thalami, thin corpus callosum, no lactatepeak in MRS	Plasma (2.8 mmol/l)	N/A
P13/F9	Bruni et al. (1)	M	Homozygous c.1100C>T	Afganistan	Alive at 5 m.	From birth	No HCM	Hypotonia	Unilateral mild cerebellar hemispheric hypoplasia	Plasma (4.4 mmol/l), CSF (3.08 mmol/l)	N/A
P14/F10	Diodato et al. (6)	M	Homozygous c.1100C>T	Italian	Alive at 8 y.	From birth	N/A	Psychomotor delay, microcephaly, epilepsy, status epilepticus	Hyperintense lesions in the periventricular regions, the insulae, and the frontotemporal right cortex, MRS: lactate peak in the frontal white matter + facial dysmorphy	N/A	↓ CI
P15/F11	San Millan et al. (8)	N/A	c.1010C>T stop codon	N/A	Death 4 m.	From birth	HCM	Floppy infant, tongue fasciculation	N/A	N/A	↓ CIV
P16/F12	Baertling et al. (11)	M	c.601C>T c.1100C>T	Greek	Alive at 5 m.	From birth	HCM	Epilepsy, burst suppression, spasticity, microcephaly, exotrophy	Hypoplasia of the corpus callosum and the cerebellum, edema of the brain stem and the frontal white matter, hyperintensities in basal ganglia displayed, MRS lactate peak	Plasma (>28 mmol/l)	N/A
P17/F13	Alsemari et al. (10)	M	Homozygous c.3650G>A	Saudi Arabia	23 y.	4 m.	N/A	Severe mental retardation, ataxia, speech impairment, epilepsy	Cerebellar atrophy + short stature, microcephaly, dysmorphia, excitable personality, excessive chewing mouth behaviors, severe growth hormone deficiency, hypogonadism, severe osteomalacia, Angelman-like syndrome	N/A	N/A
P18/F14	Ma et al. (12)	F	c.643C>T c.1354A>G	Chinese	Death at 16 d.	From birth	HCM	Poor sucking, hypertonia	The brain ultrasonic examination: mild echo enhancement on the side of the bilateral paraventricular parenchyma, a left-ependymal cyst and a right-choroid plexus cyst + PPHN	Plasma (3-15.6 mmol/l)	N/A
P19/F15	Pereira et al. (13)	F	Homozygous c.1100C>T	Portuguese	Death at 28 m.	From birth	No HCM	Microcephaly, severe global hypotonia, severe epileptic encephalopathy	Global atrophy and a small glioepithelial cyst associated with left hippocampal molding + progressive feedings difficulties, and failure to thrive	Plasma (5.35 mmol/l)	Normal
P20/F16	Ruzman et al. (14)	F	c.1100C>T c.603_606dupGATG	N/A	Death at 10 m.	1 month	HCM	Microcephaly, infantile spasms with hypsarrhytmia on EEG, later burstsuppression pattern, severe global hypotonia	Diffuse cerebral atrophy, hypoplasia of the cerebellum (vermis), brainstem, and corpus callosum, MRS high lactate peak	Plasma (2.0–5.4 mmol/l), CSF (3 mmol/l), Alanin plasma and CSF (537 and 36.1 umol/l)	N/A
P21/F17	Begliuomini et al. (15)	F	Homozygous c.1100C>T	Sardinian	Alive at 6 years	From 11 months	No HCM	Motor and language delays, hypotonia, brisk tendon reflexes	Atrophic progression of the cerebellum with T2-FLAIR hyperintensities of cerebellar white matter and dentate nuclei, MRS increased lactate and decreased N-acetyl aspartate peaks	N/A	↓ CI + CIII
P22/F18	Begliuomini et al. (15)	F	Homozygous c.1100C>T	Sardinian	Alive at 5 years	From 12months	No HCM	Nystagmus with alternating strabismus, brisk tendon reflexes, global hypotonia and impaired coordination, swallowing difficulties	Cerebellar atrophy and vermis hypoplasia with normal MRS	increased	N/A
P23/F19	Chin et al. (7)	M	c.1940C>T c.2318G>A	Chinese	Alive at 14 months	From 1 month	No HCM	Developmental delay	Normal MRI of the brain + PPHN, moderate hypertrophy of RV and dilat. RA and RV, growth failure, gastroesophageal reflux	Plasma (9.2 mmol/l)	N/A

*P/F, patient/family; F, female; M, male; d., day; m., month; y., year; HCM, hypertrophic cardiomyopathy; RV, right ventricle; RA, right atrium; PPHN, persistent pulmonary hypertension of the newborn; MRI, magnetic resonance imaging; MRS, magnetic resonance spectroscopy; CSF, cerebrospinal fluid; N/A, not available information; CI-CV, OXPHOS complex I–V. Arrow down: reduced values compared to healthy controls. Arrow up: increased values compared to healthy controls*.

## Conclusion

The data and finding of a novel variants in the *VARS2* gene of our case expands the spectrum of known mutations in humans and the spectrum of associated clinical features. Nowadays, thanks to increasing number of publish data we can assume a reduced impact of the homozygous c.1100C>T (p.Thr367Ile) mutation on the myocardium, but we cannot postulate that specific OXPHOS complexes or organ systems are impaired due to specific mutations in the *VARS2* gene. Further investigations to find specific genotype-phenotype correlations are needed in this field. It is known that biallelic mutations in the *VARS2* gene cause systemic impairment. Structural cardiac abnormalities and hypertrophic cardiomyopathy (HCM) could be the first manifestation of the disease leading to early death in the newborn period or in early infancy before developing other clinical features. That is why we think that it is of utmost importance to consider the presence of a possible mitochondriopathy in these patients and to include the analysis of the *VARS2* gene in the genetic diagnostic algorithm in cases with early manifesting and rapidly progressing HCM with hyperlactatemia.

## Data Availability Statement

Publication of the complete exome data is not included in the consent for clinical exome sequencing. The VARS2 variants have been made publicly available via ClinVar. Requests to access the datasets in more detail can be directed to the corresponding author.

The ClinVar registration numbers are: SUB9123334, SUB9123465 and they are publicly available:

Variant 1: https://www.ncbi.nlm.nih.gov/clinvar/variation/522814/

Variant 2: https://www.ncbi.nlm.nih.gov/clinvar/variation/997679/.

## Ethics Statement

The studies involving human participants were reviewed and approved by Ethics Committee of the Land Salzburg (number 415-E/2552/10-2019). Written informed consent to participate in this study was provided by the participants' legal guardian/next of kin.

## Author Contributions

This manuscript was written by KK under the guidance of DW. RF, SW, and JM conceptualized figures, revised, and edited the manuscript. H-CD performed sample collection. BC provided clinical data. OK performed muscle biopsy. All authors contributed to the article and approved the submitted version.

## Conflict of Interest

The authors declare that the research was conducted in the absence of any commercial or financial relationships that could be construed as a potential conflict of interest.

## Supplementary Material

Supplementary Table 1 Densitometric analysis of the western blotting experiments shown in Figure 2 and Supplementary Figure 1.

Supplementary Figure 1 Western blot analysis of the muscle sample of the patient compared to healthy controls, using antibodies against VARS2, NADH dehydrogenase [ubiquinone] iron-sulfur protein 4 (NDUFS4), ubiquinol-cytochrome c reductase core protein 2 (UQCRC2), succinate dehydrogenase complex flavoprotein subunit A (SDHA), ATP synthase F1 subunit alpha (ATP5F1A), voltage-dependent anion-selective channel 1 (VDAC1), citrate synthase (CS), glucosephosphate isomerase (GPI). Because of a low gel resolution ATP5F1A and UQCRC2 cannot be visually separated in the technical replicate 1 of the western blot.

Supplementary Figure 2 Immunohistochemical staining of VDAC1 and subunits of the OXPHOS complexes in skeletal muscle of affected individual and controls. **(A–C)** VDAC1 staining; **(D–F)** NDUFB8 staining; **(G–I)** SDHA staining; **(J–L)** UQCRC2 staining; **(M–O)** MT-CO1 staining; **(P–R)** ATP5F1A staining; **(A,D,G,J,M,P)** control 1; **(B,E,H,K,N,Q)** control 2; **(C,F,I,L,O,R)** affected individual. Images were taken with a 400x magnification. A slight reduction of NDUFB8 and UQCRC2 was present in muscle of the affected individual compared to controls.
