# Supplementary material for: Case Report and Review of the Literature: A New and a Recurrent Variant in the VARS2 Gene Are Associated With Isolated Lethal Hypertrophic Cardiomyopathy, Hyperlactatemia, and Pulmonary Hypertension in Early Infancy
Source: Front Pediatr. 2021 Apr 16;9:660076. doi: 10.3389/fped.2021.660076 (PMC8085550; doi:10.3389/fped.2021.660076)
Supplement: Supplementary Table 1 — Densitometric analysis of the western blotting experiments shown in Figure 2 and Supplementary Figure 1. [file Table_1.pdf]

Supplementary Table 1: Densitometric analysis of the western blotting experiments shown in Figure 2 and Supplementary Figure 1

	Controls			Patient				
Ratio	Mean	SD	n	Mean	SD	n	Relative amount (%)	P value
**Western blot- technical replicate 1**								
VARS2/VDAC1	0,5659	0,2092	n=7	0,1721	0,038	n=2	30	**0,0393**
VARS2/SDHA	0,8886	0,1701	n=7	0,1438	0,008	n=2	16	**0,0006**
VARS2/CS	1,695	0,9505	n=7	0,2684	0,008	n=2	16	0,0556
VARS2/GPI	1,457	0,5605	n=7	0,5017	0,283	n=2	34	0,0594
**Mean reduction of VARS2 protein: 76%**								
NDUFS4/VDAC1	1,393	0,5595	n=7	0,9939	0,446	n=2	71	0,3914
NDUFS4/SDHA	0,8981	0,381	n=7	0,2948	0,022	n=2	33	0,0704
NDUFS4/CS	1,681	1,011	n=7	0,5513	0,056	n=2	33	0,1763
NDUFS4/GPI	0,5781	0,3764	n=7	0,349	0,03	n=2	60	0,4394
**Mean reduction of NDUFS4 protein: 51%**								
**Western blot- technical replicate 2**								
NDUFS4/VDAC1	0,8682	0,3252	n=3	0,2622	n.a	n=1	30	n.a
NDUFS4/SDHA	0,5028	0,2419	n=3	0,0857	n.a	n=1	17	n.a
NDUFS4/CS	0,5631	0,2685	n=3	0,1778	n.a	n=1	32	n.a
NDUFS4/GPI	3,42	1,098	n=3	1,798	n.a	n=1	53	n.a
**Mean reduction of NDUFS4 protein: 67%**								
SDHA/VDAC1	1,822	0,3001	n=3	3,079	n.a	n=1	169	n.a
SDHA/CS	1,147	0,1639	n=3	2,087	n.a	n=1	182	n.a
SDHA/GPI	7,709	3,015	n=3	21,11	n.a	n=1	274	n.a
UQCRC2/VDAC1	1,329	0,341	n=3	0,6968	n.a	n=1	52	n.a
UQCRC2/SDHA	0,7514	0,2754	n=3	0,2263	n.a	n=1	30	n.a
UQCRC2/CS	0,8636	0,3532	n=3	0,4724	n.a	n=1	55	n.a

UQCRC2/GPI	5,255	0,4552	n=3	4,778	n.a	n=1	91	n.a
MT-CO2/VDAC1	0,4265	0,1288	n=3	0,2428	n.a	n=1	57	n.a
MT-CO2/SDHA	0,2371	0,0812	n=3	0,0792	n.a	n=1	33	n.a
MT-CO2/CS	0,2636	0,0503	n=3	0,1652	n.a	n=1	63	n.a
MT-CO2/GPI	1,853	0,925	n=3	1,671	n.a	n=1	90	n.a
ATP5F1A/VDAC1	2,444	0,3096	n=3	3,589	n.a	n=1	147	n.a
ATP5F1A/SDHA	1,35	0,1308	n=3	1,166	n.a	n=1	86	n.a
ATP5F1A/CS	1,534	0,0808	n=3	2,433	n.a	n=1	159	n.a
ATP5F1A/GPI	10,38	3,906	n=3	24,61	n.a	n=1	237	n.a

Ratio: Ratio of the band intensities of western blot analysis. SD: standard deviation; n.a: not applicable
