# Supplementary figures and images for: Case Report and Review of the Literature: A New and a Recurrent Variant in the VARS2 Gene Are Associated With Isolated Lethal Hypertrophic Cardiomyopathy, Hyperlactatemia, and Pulmonary Hypertension in Early Infancy
Source: Front Pediatr. 2021 Apr 16;9:660076. doi: 10.3389/fped.2021.660076 (PMC8085550; doi:10.3389/fped.2021.660076)

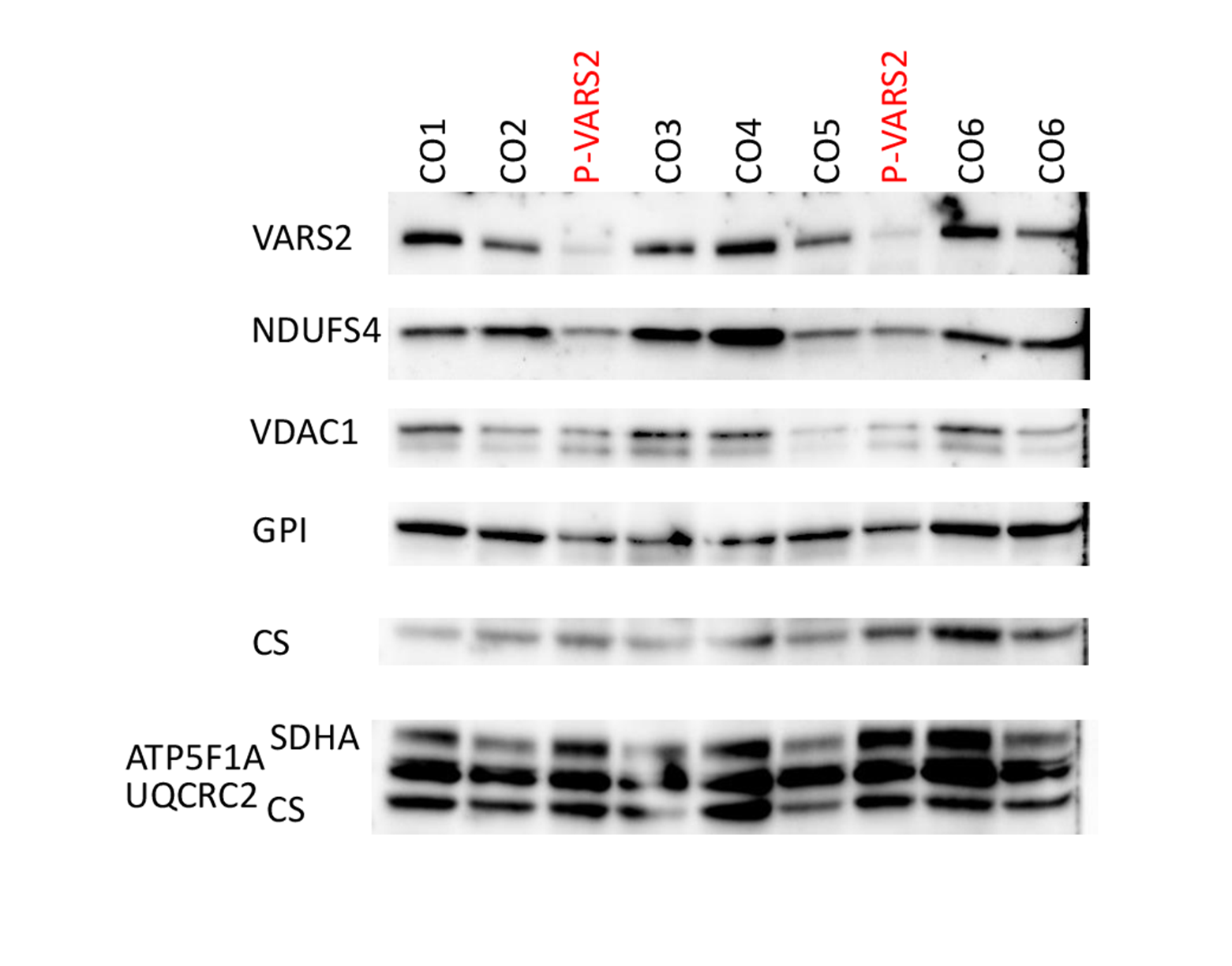

Supplement: Supplementary Figure 1 — Western blot analysis of the muscle sample of the patient compared to healthy controls, using antibodies against VARS2, NADH dehydrogenase [ubiquinone] iron-sulfur protein 4 (NDUFS4), ubiquinol-cytochrome c reductase core protein 2 (UQCRC2), succinate dehydrogenase complex flavoprotein subunit A (SDHA), ATP synthase F1 subunit alpha (ATP5F1A), voltage-dependent anion-selective channel 1 (VDAC1), citrate synthase (CS), glucosephosphate isomerase (GPI). Because of a low gel resolution ATP5F1A and UQCRC2 cannot be visually separated in the technical replicate 1 of the western blot. [file Image_1.tif]

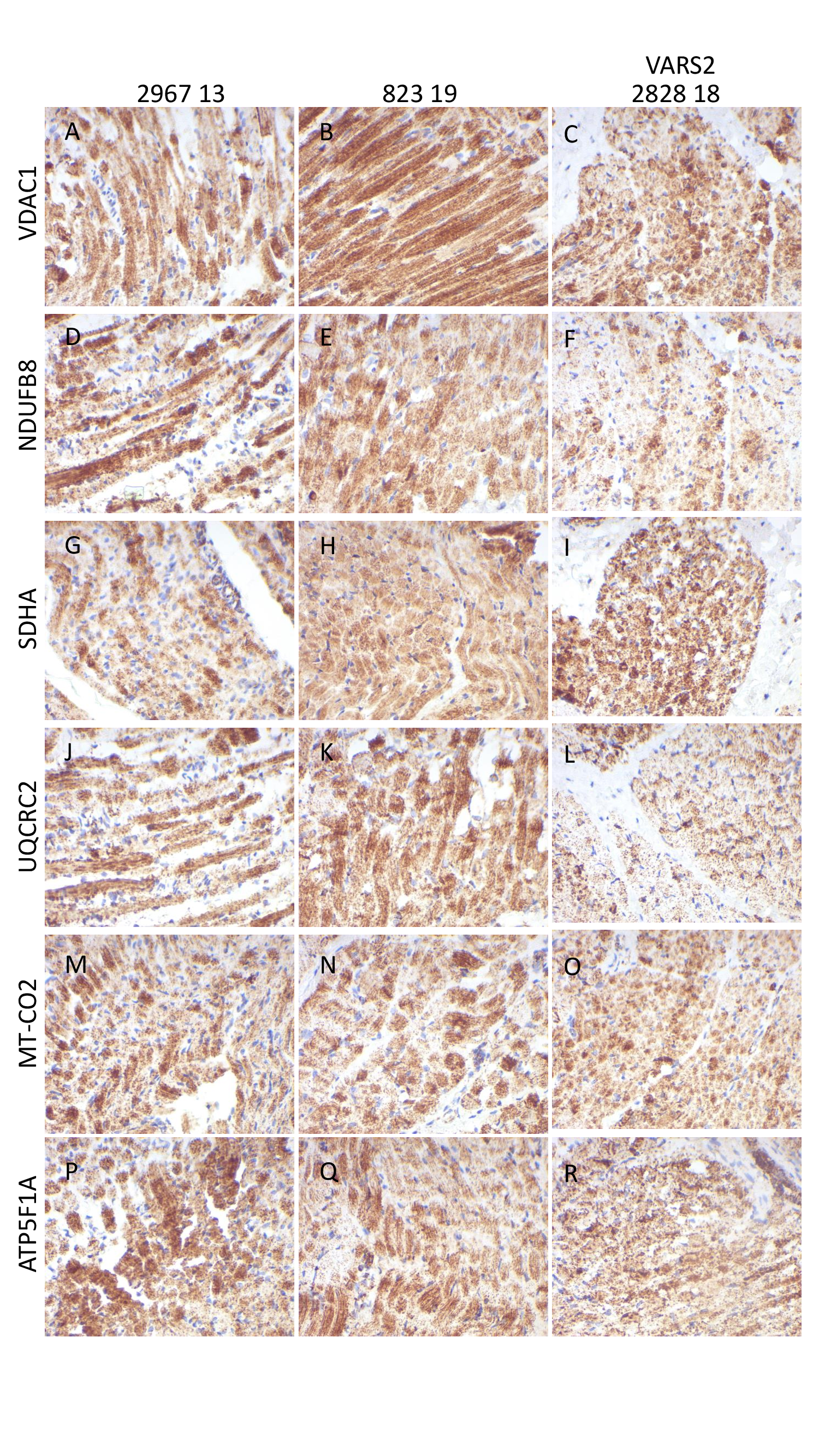

Supplement: Supplementary Figure 2 — Immunohistochemical staining of VDAC1 and subunits of the OXPHOS complexes in skeletal muscle of affected individual and controls. (A–C) VDAC1 staining; (D–F) NDUFB8 staining; (G–I) SDHA staining; (J–L) UQCRC2 staining; (M–O) MT-CO1 staining; (P–R) ATP5F1A staining; (A,D,G,J,M,P) control 1; (B,E,H,K,N,Q) control 2; (C,F,I,L,O,R) affected individual. Images were taken with a 400x magnification. A slight reduction of NDUFB8 and UQCRC2 was present in muscle of the affected individual compared to controls. [file Image_2.tiff]
